# Autogenous *Escherichia coli* Vaccine Application as an Innovative Antimicrobial Therapy in Poultry Farming—A Case Report

**DOI:** 10.3390/vaccines10091567

**Published:** 2022-09-19

**Authors:** Liča Lozica, Céline Sadaf Morteza Gholi, Adaya Kela, Ivan Lošić, Danijela Horvatek Tomić, Željko Gottstein

**Affiliations:** 1 Department of Poultry Diseases with Clinic, Faculty of Veterinary Medicine, University of Zagreb, Heinzelova 55, 10000 Zagreb, Croatia; 2 Faculty of Veterinary Medicine, University of Zagreb, Heinzelova 55, 10000 Zagreb, Croatia

**Keywords:** *Escherichia coli*, colibacillosis, poultry, vaccination, autogenous vaccine, innovative antimicrobial therapy

## Abstract

*Escherichia coli (E. coli)* is one of the most common bacterial causes of infection in poultry farming. Whether the infection is localized or systemic, a primary or secondary disease, it is most frequently treated through the application of wide-spectrum antimicrobials. Excessive use of antimicrobials in agriculture is significantly contributing to the worldwide rise of antimicrobial resistance, but is also very expensive and often ineffective in the long term. Here, we present a case where a colibacillosis outbreak on a family farm of laying hens was treated using an autogenous vaccine. The birds had septicemia, cellulitis, and severe skin wounds. They were not vaccinated against *E. coli,* and did not receive any antimicrobials previously. *E. coli* strains were isolated from the daily mortalities on the farm and used for preparation of the vaccine. Each bird was given an intramuscular injection of the autogenous vaccine. The immunogenicity of the vaccine was tested by the determination of specific antibody levels in the sera of the birds using the in-house ELISA. Shortly after vaccination, the morbidity and mortality rates significantly decreased, and egg production was improved. The application of the autogenous vaccine served as a curative and preventive measure, and has proven to be a very efficient method of antimicrobial therapy.

## 1. Introduction

Colibacillosis is one of the most common bacterial diseases in poultry farming. The infection can be localized, but more regularly it is systemic and leads to high mortality on farms [1,2]. The lesions in adult egg-laying hens most frequently include peritonitis, polyserositis, and salpingitis [3,4]. Whether the infection is primary or secondary, the outbreaks are managed by the application of antimicrobials through the drinking water [5]. The treatment is usually based on the application of wide-spectrum antimicrobials, often without any susceptibility testing beforehand. Consequently, that kind of excessive and inadequate application leads to increasing levels of resistance through a gradual selection of the highly virulent and resistant *Escherichia coli (E. coli)* strains [6,7]. Although the use of antimicrobials seems like the most efficient and quickest solution, they act non-selectively and affect the entire microbiota [8]. Moreover, the application of antimicrobials leads to the accumulation of antimicrobial resistance in the bacterial population and possible transmission of resistance genes to the human population as consumers of various poultry products [2,7]. As the overall consumption of antimicrobials in agricultural production in Croatia is often overlooked, it is necessary to conduct continuous monitoring of the common bacterial pathogens on farms and explore the possibilities of alternative and innovative antimicrobial therapies.

The control and prevention of colibacillosis on farms includes continuous monitoring, strict biosecurity measures, vaccination, and the application of antimicrobials upon indication [5,7]. Other possible alternative strategies include prevention by competitive exclusion, the use of probiotics or phytogenic additives, phage therapy, and vaccination as a treatment method [7,9,10,11,12,13,14]. Vaccination is usually associated with the prevention of the disease, but it can also be used as a curative method. Some of the biggest challenges in the selection of a vaccine against *E. coli* is the immense heterogeneity and poor cross-protection between different strains in the field [5,15]. However, the application of autogenous vaccines enables the selection of strains homologous to the ones involved in the colibacillosis outbreak [16,17] and can be used as an innovative treatment method [18].

Here, we present a case of colibacillosis outbreak on a family farm of laying hens that was resolved using autogenous vaccine as an alternative strategy of antimicrobial therapy.

## 2. Material and Methods

### 2.1. Case History

A flock of 600 36-week-old laying hens on a free-range family farm showed lesions indicative of colibacillosis. The lesions included fibrinous peritonitis, perihepatitis, polyserositis, splenomegaly, and cellulitis. Due to an avian influenza lockdown at the time of the outbreak, the birds were held inside a semi-open poultry house, in a floor production system. The housing system included three areas with vertical perches, nests, and waterers and feeders, respectively. The area with feed and water access had no litter, and was cleaned daily using a water hose. The birds were vaccinated as pullets according to the recommended vaccination program, but they were not previously vaccinated against *E. coli.* The farmers noticed several aggressive birds in the flock, but did not detect major signs of cannibalism. During one month since the birds started showing symptoms, the morbidity and mortality rates were high and continuously increasing, while the egg production significantly declined. As the affected flock was at the peak of production and had already suffered major losses, the farmers were advised to apply an autogenous *E. coli* vaccine in order to stop further development of the outbreak and to evade the need for antimicrobial treatment.

### 2.2. Sampling and Laboratory Testing

After the clinical examination of the birds, blood samples were collected for serological testing. Ten birds were randomly selected and sampled before and 21 days after the vaccination procedure. Five carcasses were pathomorphologically examined and swab samples were collected from the peritoneum, liver, bone marrow, and other macroscopically changed organs upon indication. The swabs were streaked directly onto MacConkey agar (Oxoid, Basingstoke, UK), UTI Brilliance Clarity Chromogenic agar (Oxoid, Basingstoke, UK), and Columbia agar (Rapid Labs, Colchester, UK) enriched with 5% sheep blood (Biognost, Zagreb, Croatia) for bacteriological examination. All plates were aerobically incubated at 37 °C for 24 h. The identification of the bacteria was based on the macroscopic morphological characteristics of the colonies and biochemical analyses. All bacterial strains were purified and stored in brain–heart infusion broth (Biolife, Italy, Milan) at –20 °C until the vaccine preparation.

### 2.3. Antimicrobial Susceptibility Testing

Antimicrobial susceptibility testing was routinely performed for all isolated *E. coli* samples using disk diffusion assay on Müller–Hinton agar (Oxoid, Basingstoke, UK). The inoculum was prepared by suspending 24-h-old bacterial colonies in sterile saline solution and adjusting to turbidity of 0.5 McFarland standards. The testing was performed with the following antibiotic discs—10 µg of amoxicillin, 30 µg of doxycycline, 5 µg of enrofloxacin, 30 µg of florfenicol, 109 µg of lincomycin/spectinomycin, 10 µg of norfloxacin, and 25 µg of trimethroprim/sulfamethoxazole (Oxoid, Basingstoke, UK). After overnight aerobic incubation at 37 °C, the inhibition zone diameters were interpreted according to the European Committee on Antimicrobial Susceptibility Testing (EUCAST) and the British Society for Antimicrobial Chemotherapy (BSAC) guidelines.

### 2.4. DNA Extraction and Phylogenetic Group Analysis

DNA extraction for the polymerase chain reaction (PCR) was performed using the boiling method. Several colonies of each *E. coli* strain were aseptically collected, put in a 1.5 mL tube containing 150 μL of nuclease-free water (Promega, Madison, WI, USA), boiled for 20 min in a thermoblock, and then centrifuged for 10 min at 13,000 rpm. The supernatant containing the DNA was separated and used for PCR analysis. Phylogenetic groups were determined using the adapted quadruplex PCR protocol developed by Clermont et al. (2013) [19], as described by Lozica et al. (2021a) [20].

### 2.5. Vaccine Preparation and Vaccination

The birds were vaccinated with a specifically manufactured vaccine prepared using *E. coli* strains isolated from the bone marrow of the deceased birds (Table 1) and four highly virulent strains of different phylogroups that have been frequently detected as causative agents of colibacillosis on Croatian poultry farms. The selected strains from the farm were revived on UTI Brilliance Clarity chromogenic agar (Oxoid, Basingstoke, UK) and then amplified in the brain–heart infusion broth (Biolife, Italy, Milan) at 37 °C for 24 h for the vaccine preparation. The bacteria were then purified by sedimentation through the centrifugation process. The supernatant was removed and the pellet was additionally washed twice using sterile saline solution. The antigen was processed using an ultrasound probe KE76 (Sonoplus HD 2200, Bandelin, Germany) under 20 kHz for 30 min. The homogenized antigen was inactivated in a water bath at 95 °C for 1 h. The sterility was tested by plating on Columbia blood agar and incubated in aerobic and anaerobic conditions at 37 °C for 48 h. The prepared antigen was then mixed with mineral oil (Montanide™ ISA 71 R VG, Seppic, Paris, France) to create an oil–emulsion-based inactivated vaccine. Each bird received 0.3 mL of the vaccine with the bacterial concentration of 5 × 10^8^ CFU/dose injected intramuscularly.

### 2.6. Enzyme-Linked Immunosorbent Assay

The blood samples were transferred to 2 mL tubes and left to clot for approximately 1 h. Afterwards, the sera were separated, transferred to clean tubes, and kept at −20 °C until the serological analysis. In order to test the immunogenicity of the vaccine, the samples were analyzed using the direct in-house enzyme-linked immunosorbent assay (ELISA) according to the protocol described by Leitner et al. (1990) [21]. The homogenized antigen, which was used for the vaccine preparation, was also used as antigen for ELISA. The plate was read using μQuant microplate spectrophotometer (BioTek Instruments, Inc., Winooski, VT, USA) at 405 nm.

### 2.7. Statistical Analysis

The statistical analysis was performed in Statistica 13.5.0.17. (TIBCO Software Inc., Tulsa, OK, USA). The results were tested for data distribution normality using the Kolmogorov–Smirnov test, while the significance of difference in the OD values was analyzed using a Wilcoxon test with statistical significance set at the level of *p* < 0.05.

## 3. Results and Discussion

Clinical examination showed that all birds in the flock had skin lesions around the cloaca, indicating a severe case of cannibalism, and used standard feed and water regularly, without significant weight loss. Gross pathology observations included fibrinous peritonitis, perihepatitis, polyserositis, splenomegaly, and severe cellulitis. As suspected, *E. coli* was isolated in high quantities from all swab samples except one liver sample. There were no signs indicating other possible diseases. The presence of the bacteria in the spleen, liver, and bone marrow indicated the birds had systemic infection and septicemia, i.e., colisepticemia, which is considered a disease of pullets triggered by stress [22].

One of the methods that are currently often used for typing of *E. coli* strains is the phylotyping method according to the protocol described by Clermont et al. (2013, 2019) [19,23]. This method has proven to be very efficient for the detection of highly virulent strains (B2, D, F) and the assessment of strain diversity in the field [24,25,26]. Here, all analyzed strains belonged to phylogenetic group C (Table 1), which is considered a sister-group to the B1 phylogenetic commensal group [24].

An antimicrobial susceptibility assay showed all strains were resistant to doxycycline, while 57.14% of the strains were resistant to amoxicillin (Table 2). Although the owners did not report using antimicrobials in this flock, the application of antimicrobial treatment in the previous flocks and the circulation of resistant bacteria in the environment could have affected the current resistance rates [27]. Considering the high homogeneity between *E. coli* isolates from different organs of the same bird and isolates from different birds in the same flock, the most plausible source of infection was the environment. In order to maintain the zoohygienic conditions on the farm, the owner was regularly washing the feeding and drinking area of the poultry house. The wet and warm environment created optimal conditions for bacterial growth and transmission, while skin lesions caused by cannibalism enabled the entry of bacteria and led to a colibacillosis outbreak. Therefore, the farmers were advised to reduce the frequency of washing in said area, and close it off after washing until it completely dries before letting the birds inside.

Cannibalism is the most severe form of feather and vent pecking [28]. It is stimulated by bright light, high-density housing, the absence of foraging, nutritional deficiencies, or ectoparasites, and it is usually expressed by dominant birds in the flock [29,30]. Vent pecking commonly starts at the beginning of lay, and it can trigger the onset of salpingitis and egg peritonitis, which are some of the most common lesions of colibacillosis [31]. Some conventional laying hen phenotypes are considered more prone to feather pecking [32]. In this case, as the birds were held inside due to influenza lockdown, the lack of foraging and overall stress caused by changed housing conditions probably triggered the pecking behavior.

**Figure 1:**
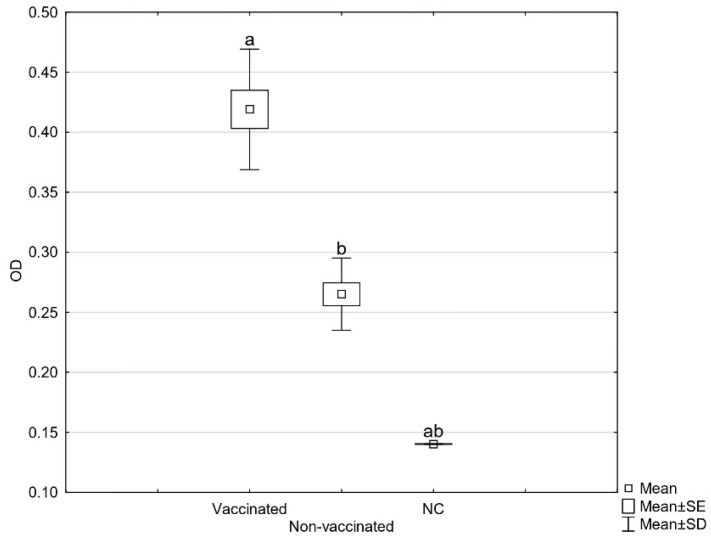
Average OD values of sera tested against *E. coli* strains used in the autogenous vaccine detected by in-house ELISA before and three weeks after vaccination (NC = negative control). Statistically significant differences in OD values are marked with different letters (a, b).

Since the beginning of the outbreak, there were 22 deaths. In the three-month period after the vaccination, the mortality rate decreased and there were ten deaths in total, of which most occurred in the first days after the vaccination due to severe systemic infections of the sick birds. As the lesions of the daily mortalities were severe, we presumed the autogenous vaccine would only be effective for the birds at the beginning of an infection or with a localized infection. In the two-week period after the vaccination, the egg production normalized, the skin lesions healed, and the mortality rate decreased. The results of the in-house ELISA showed a significant increase of specific serum antibodies three weeks after vaccination (Figure 1). The same results were observed on other laying hen farms, where ad hoc vaccination following the clinical manifestation resulted in a higher total number of produced eggs, declined mortality, lower contamination of the eggs, and reduced horizontal transmission of pathogenic strains [18].

The recovery of the birds on this farm was probably a result of both vaccination and the change of the housing conditions, which helped slow down the morbidity rate. Considering the cost of treatment and overall effect of vaccination on the birds’ welfare and production, the application of the autogenous *E. coli* vaccine as a treatment during an outbreak showed great results and can be recommended as an antimicrobial therapy. Very important benefits of autogenous vaccine application and the evasion of antimicrobial treatment are loss of the withdrawal period and no egg contamination, which can have a significant economic influence. Besides the treatment of the ongoing infection, the vaccination will serve as a preventive measure for the protection of the birds until the end of their production cycle. Previous research showed that the application of an autogenous vaccine in broiler breeder flocks can induce the genetic homogenization of the strains and reduce the prevalence of antimicrobial resistance genes [26,33]. Accordingly, the application of the autogenous vaccine on this farm is also expected to have a positive impact on the antimicrobial resistance rates.

## 4. Conclusions

The application of an autogenous vaccine in laying hens during production has proven to be a very efficient method of antimicrobial therapy. The vaccination significantly reduced the morbidity and mortality rates, improved egg production, and provided protection for the birds until the end of the production cycle.

## Figures and Tables

**Table 1:** Description of *E. coli* strains isolated from the daily mortalities.

Isolate	Origin		Phylogenetic Group
	Bird	Organ	
2112	1	liver	C
2113	1	subcutaneous tissue ^a^	C
2114	1	peritoneum	C
2115	1	spleen	C
2116 ^b^	1	bone marrow	C
2117	2	subcutaneous tissue	C
2118	2	spleen	C
2119	2	peritoneum	C
2120	2	air sacs	C
2121 ^b^	2	bone marrow	C
2122	3	liver	C
2123 ^b^	3	bone marrow	C
2124	3	spleen	C
2125	3	peritoneum	C
2126	4	subcutaneous tissue	C
2127	4	spleen	C
2128	4	oviduct	C
2129	4	liver	C
2130 ^b^	4	bone marrow	C
2131	5	peritoneum	C
2132	5	subcutaneous tissue	C

^a^ Subcutaneous tissue underneath the skin lesions (cellulitis). ^b^ Strains used for the preparation of the vaccine.

**Table 2:** Results of the antimicrobial susceptibility assay showing the number (%) of susceptible (S), intermediate (I) or resistant (R) strains to each tested antimicrobial agent.

	Antimicrobial Agent						
	AML ^a^ (10 μg)	DO (30 μg)	ENR (5 μg)	FFC (30 μg)	LS (109 μg)	NOR (10 μg)	SXT (25 μg)
S	9 (42.86)	-	21 (100)	21 (100)	17 (80.95)	21 (100)	21 (100)
I	-	-	-	-	4 (19.05)	-	-
R	12 (57.14)	21 (100)	-	-	-	-	-

^a^ AML—amoxicillin, DO—doxycycline, ENR—enrofloxacin, FFC—florfenicol, LS—lincomycin/spectinomycin, NOR—norfloxacin, SXT—trimethoprim/sulfamethoxazole.

## References

[1] Nolan L.K., Vaillancourt J.-P., Barbieri N.L., Logue C.M., Swayne D.E. (2020). Colibacillosis. Diseases of Poultry.

[2] Mageiros L., Méric G., Bayliss S.C., Pensar J., Pascoe B., Mourkas E., Calland J.K., Yahara K., Murray S., Wilkinson T.S. (2021). Genome evaluation and the emergence of pathogenicity in avian *Escherichia coli*. Nat. Commun..

[3] Pires Dos Santos T., Bisgaard M., Christensen H. (2013). Genetic diversity and virulence profiles of *Escherichia coli* causing salpingitis and peritonitis in broiler breeders. Vet. Microbiol..

[4] Li L., Thøfner I., Christensen J.P., Ronco T., Pedersen K., Olsen R.H. (2017). Evaluation of the efficacy of an autogenous *Escherichia coli* vaccine in broiler breeders. Avian Pathol..

[5] Koutsianos D., Gantelet H., Franzo G., Lecoupeur M., Thibault E., Cecchinato M., Koutoulis K.C. (2020). An assessment of the level of protection against colibacillosis conferred by several autogenous and/or commercial vaccination programs in conventional pullets upon experimental challenge. Vet. Sci..

[6] Ievy S., Islam M.S., Sobur M.A., Talukder M., Rahman M.B., Khan M.F.R., Rahman M.T. (2020). Molecular detection of avian pathogenic *Escherichia coli* (APEC) for the first time in layer farms in Bangladesh and their antibiotic resistance patterns. Microorganisms.

[7] Christensen H., Bachmeier J., Bisgaard M. (2021). New strategies to prevent and control avian pathogenic *Escherichia coli* (APEC). Avian Pathol..

[8] Kairmi S.H., Taha-Abdelaziz K., Yitbarek A., Sargolzaei M., Spahany H., Astill J., Shojadoost B., Alizadeh M., Kulkarni R.R., Parkinson J. (2022). Effects of therapeutic levels of dietary antibiotics on the cecal microbiome composition of broiler chickens. Poul. Sci..

[9] Oliveira A., Sereno R., Azaredo J. (2010). In vivo efficiency evaluation of a phage cocktail in controlling severe colibacillosis in confined conditions and experimental poultry houses. Vet. Microbiol..

[10] Abd-El-Ghany W.A., Ismail M. (2014). Tackling experimental colisepticaemia in broiler chickens using phytobiotic essential oils and antibiotic alone or in combination. Iran. J. Vet. Res..

[11] Ceccarelli D., Van-Essen-Zandbergen A., Smid B., Veldman K.T., Boender G.J., Fischer E.A.J., Mevius D.J., Van Der Goot J.A. (2017). Competitive exclusion reduces transmission and excretion of extended-spectrum-β-lactamase-producing *Escherichia coli* in broilers. Appl. Environ. Microbiol..

[12] Wernicki A., Nowaczek A., Urban-Chmiel R. (2017). Bacteriphage therapy to combat bacterial infections in poultry. Virol. J..

[13] Chantziaras I., Smet A., Filippitzi M.E., Damiaans B., Haesebrouck F., Boyen F., Dewulf J. (2018). The effect of a commercial competitive exclusion product on the selection of enrofloxacin resistance in commensal *E. coli* in broilers. Avian Pathol..

[14] Methner U., Rösler U. (2020). Efficacy of a competitive exclusion culture against extended-spectrum ß-lactamase-producing *Escherichia coli* strains in broiler using a seeder bird model. BMC Vet. Res..

[15] Guabiraba R., Schouler C. (2015). Avian colibacillosis: Still many black holes. FEMS Microbiol. Lett..

[16] Landman W.J.M., Van Eck J.H.H. (2017). The efficacy of inactivated *Escherichia coli* autogenous vaccines against the *E. coli* peritonitis syndrome in layers. Avian Pathol..

[17] Kromann S., Olsen R.H., Bojesen A.M., Jensen H.E., Thøfner I. (2021). Protective potential o fan autogenous vaccine in an aerogenous model of *Escherichia coli* infection in broiler breeders. Vaccines.

[18] Gottstein Ž., Lozica L., Lukač M., Horvatek Tomić D. Production parameters after ad hoc application of autogenous vaccine during production following clinical colibacillosis in layer flocks. Proceedings of the XIV Symposium Poultry Days 2022.

[19] Clermont O., Christenson J.K., Denamur E., Gordon D.M. (2013). The Clermont *Escherichia coli* phylo-typing method revisited: Improvement of specificity and detection of new phylo-groups. Environ. Microbiol. Rep..

[20] Lozica L., Ekert Kabalin A., Dolenčić N., Vlahek M., Gottstein Ž. (2021). Phylogenetic characterization of avian pathogenic *Escherichia coli* strains longitudinally isolated from broiler breeder flocks vaccinated with autogenous vaccine. Poult. Sci..

[21] Leitner G., Melamed D., Drabkin N., Heller E.D. (1990). An enzyme-linked immunosorbent assay for detection of antibodies against *Escherichia coli:* Association between indirect hemagglutination test and survival. Avian Dis..

[22] Zanella A., Alborali G.L., Bardotti M., Candotti P., Guadagnini P.F., Martino P.A., Stonfer M. (2000). Severe *Escherichia coli* O111 septicaemia and polyserositis in hens at the star of lay. Avian Pathol..

[23] Clermont O., Dixit O.V.A., Vangchhia B., Condamine B., Dion S., Bridier-Nahmias A., Denamur E., Gordon D. (2019). Characterization and rapid identification of phylogroup G in *Escherichia coli,* a lineage with high viurlence and antibiotic resistance potential. Environ. Microbiol..

[24] Logue C.M., Wannemuehler Y., Nicholson B.A., Doetkott C., Barbieri N.L., Nolan L.K. (2017). Comparative analysis of phylogenetic assignment of human and avian ExPEC and fecal commensal *Escherichia coli* using the (previous and revised) Clermont phylogenetic typing methods and its impact on avian pathogenic *Escherichia coli* (APEC) classification. Front. Microbiol..

[25] Starčič Erjavec M., Predojević L., Žgur-Bertok D. (2017). Commentary: Comparative analysis of phylogenetic assignment of human and avian ExPEC and fecal commensal *Escherichia coli* using the (previous and revised) Clermont phylogenetic typing methods and its impact on avian pathogenic *Escherichia coli* (APEC) classification. Front. Microbiol..

[26] Lozica L., Repar J., Gottstein Ž. (2021). Longitudinal study on the effect of autogenous vaccine application on the sequence type and virulence profiles of *Escherichia coli* in broiler breeder flocks. Vet. Microbiol..

[27] Hess C., Troxler S., Jandreski-Cvetkovic D., Zloch A., Hess M. (2022). *Escherichia coli* isolated form organic laying hens reveal a high level of antimicrobial resistance despite no antimicrobial treatments. Antibiotics.

[28] Turner S.P. (2011). Breeding against harmful social behaviours in pigs and chickens: State of the art and the way forward. Appl. Anim. Behav. Sci..

[29] Crespo R., Swayne D.E. (2020). Developmental, metabolic, and other noninfectious disorders. Diseases of Poultry.

[30] El-Lethey H., Aerni V., Jungi T.W., Wechsler B. (2000). Stress and feather pecking in laying hens in relation to housing conditions. Br. Poult. Sci..

[31] Gray P., Jenner R., Norris J., Page S., Browning G., Australian Veterinary Association Ltd., Animal Medicines Australia (2021). Antimicrobial prescribing guidelines for poultry. Aust. Vet. J..

[32] Giersberg M.F., Spindler B., Rodenburg B., Kemper N. (2020). The dual purpose hen as a chance: Avoiding injurious pecking in modern laying hen husbandry. Animals.

[33] Lozica L., Villumsen K.R., Li G., Hu X., Maurić Maljković M., Gottstein Ž. (2022). Genomic analysis of *Escherichia coli* longitudinally isolated from broiler breeder flocks after application of an autogenous vaccine. Microorganisms.

